# 

**Published:** 1919-09-20

**Authors:** 


### THE COLLEGE OF NURSING.

### Its Coat of Arms and Badge: An Attractive Competition.

We again remind our readers that it is proposed to apply for a Coat of Arms and Badge for the College of Nursing, and it is thought that members of the College and those interested in it may desire to express their views as to the emblems and mottoes which might most suitably be adopted as indicative of the work and objects of the College.

In order to ascertain these views the Council has accepted, with grateful thanks the offer of The Hospital of a prize of £10 for the best design for a Coat of Arms, and the College offers a first prize of £7 10s., and a second prize of £2 10s., for the best design for a Badge, with a view to these being submitted, if thought suitable, to the proper authorities. Competitors are invited to send in their designs in a covered envelope marked "Coat of Arms" and "Badge" respectively.

Simplicity in design should be aimed at in both, which should be suitable to be borne on either nurses' uniforms or on the property of the College generally.

The last day for sending in designs is September 30, 1919. But the designs will be received on or before September 30, and all should be addressed to the Secretary, the College of Nursing, Ltd., 7 Henrietta Street, Cavendish Square, W. 1.

### PASSING EVENTS AND TOPICS.

### Lectures for Intending Tuberculosis Officers.

A COURSE of lectures for candidates desirous of obtaining positions as tuberculosis officers, for general practitioners and others will be given in October, November, and December on Thursdays, at 5 P.M., at the Royal Institute of Public Health. Further particulars can be obtained from the Secretaries, 37 Russell Square, W.C. 1.

### Islington War Memorial Fund.

THE Masonic Service, in aid of the Borough of Islington War Memorial Fund for the extension of the Great Northern Central Hospital, will be held at the Parish Church of St. Mary Islington, Upper Street, N. 1, on Sunday, November 2, in the afternoon. The preacher will be the Bishop of Chelmsford.

### Matrons' and Sisters' Appointments.

Children's Sanatorium, Holt, Norfolk.—Miss Evelyn Large has been appointed sister. She was trained at the Norfolk and Norwich Hospital, where she was later sister of the out-patient department and on the private staff. She has been a sister at the Children's Hospital, Leasowe, Cheshire.

Middleton-in-Wharfedale Sanatorium.—Miss Gertrude E. Sharpe has been appointed home sister. She was trained at the Royal Infirmary, Sunderland, and has been sister at the Middlesbrough Sanatorium; home sister at the Baguley Sanatorium, Cheshire, and the Tuberculosis Dispensary, Middlesbrough; matron of the Mirfield War Hospital.

Isolation Hospital, Ashford, Kent.—Miss E. H. Howe has been appointed charge sister. She was trained at the Eastern Hospital, Homerton, where she was staff nurse. She has been charge nurse at the Hertford and Ware Joint Hospital; sister at the Isolation Hospital, Biggleswade.

Swinton House and Parkfield Residential Schools for Crippled Children, Manchester.—Miss Frances Smith has been appointed matron. She was trained at the General Infirmary, Stockport, and the Seacroft Fever Hospital, Leeds. She has been sister at the Edinburgh Sick Children's Hospital and at the Infirmary, Ashton-under-Lyne, and housekeeping sister at the Royal Hospital for Sick Children, Glasgow. She has also done military nursing in France.

South-Eastern Hospital for Children, Sydenham, S.E.—Miss M. Coakley has been appointed night sister. She was trained at the Southwark Infirmary, East Dulwich, and has been staff nurse and sister at the Southwark Military Hospital.

Township Infirmary, Leeds.—Miss Effie Brown has been promoted from second assistant matron to the position of assistant matron. She was trained at St. Pancras Infirmary, London, N., and has been ward, theatre, night and home sister at St. Luke's War Hospital, Halifax; also home sister at the Leeds Township Infirmary. She holds the C.M.B. certificate and is a member of the College of Nursing.

City Hospital (Fever), Coventry.—Miss M. Dugdale has been appointed night sister. She was trained at Salford Union Infirmary, where she was later staff nurse, and has been staff nurse and sister at the Eastern Fever Hospital, Homerton, and night sister at Huddersfield War Hospital. Miss Dugdale has also served in Queen Alexandra's Imperial Military Nursing Service Reserve, and has done private nursing.

Fever Hospital, Tollemache Road, Birkenhead.—Miss E. S. Allen has been appointed night sister. She was trained at the Taunton and Somerset Hospital, and received her fever training at Ruchill Hospital, Glasgow. She has done military nursing under the Q.A.I.M.N.S.

Westmorland County Hospital, Kendal.—Miss S. Ellen Davies has been appointed matron. She was trained at the Liverpool Royal Infirmary, and has since been night sister at the Infirmary, Bolton, and assistant matron at the General Hospital, Northampton.

Mary Hewetson Hospital, Keswick.—Miss Lilian Ellis, A.R.R.C., has been appointed nurse-matron. She was trained at the Liverpool Royal Infirmary, where she was later sister-in-charge of the O.P. Department.

Cliff College Training Home and Mission.—Miss Ella Thorne has been appointed nurse-matron. She was trained at the North Devon Infirmary, Barnstaple. She has since been staff nurse at the Royal Sussex County Hospital, Brighton, and done private nursing. She is a member of the College of Nursing.

Midland Counties Institution for the Feeble-minded, Knowle, Birmingham.—Miss K. L. Morrall has been appointed matron. She was trained at Guy's Hospital and the Mount Vernon Hospital, Northwood. She has done private nursing, and during the war military and Red Cross work, and has been matron of the Air-raid Home for Boys, Seaford.

### The College of Nursing (Limited by Guarantee).

NOTTINGHAM CENTRE.

*Thursday, October* 16, 3 p.m.—Sir Arthur Stanley will deliver an address on "The Nation's Tribute Fund" in the Exchange Hall, Nottingham, kindly lent by the Mayor, who will preside at the meeting.

LIVERPOOL CENTRE.

*Monday, September* 22, 7 p.m.—A members' meeting will be held at the Royal Infirmary, Liverpool. The matters to be discussed are "Uniform," and the "Constitution of Local Centres."

### Forthcoming Events.

Kensington Infirmary.

**Annual Reunion, Friday, September 26.**—There will be a service in the Church of St. Elizabeth at 3.30 p.m., followed by a reception. Medals and awards will be presented by Lady Fleming. All nurses who have been trained at Kensington will be welcome.

**Harvest Thanksgiving Service, Sunday, September 28**, in the Church of St. Elizabeth, 4 p.m. The sermon will be preached by the Chaplain of St. Thomas's Hospital. The Rev. A. Lombardini will welcome friends to this service.

### Gifts and Bequests.

The Treasurers of Middlesex Hospital have received from Mrs. D. Lionel Beddington £1,050 to endow a bed in memory of her father-in-law.

Dr. H. J. Strong, J.P., of Worthing, formerly a surgeon in Croydon, left estate valued at £47,750. Subject to the payment of a few legacies the property is to be divided into twenty parts, six are given to the Royal Medical Benevolent College, Epsom, two to the Croydon General Hospital, and one to the Worthing Hospital.

Under the will of Mr. T. D. Evans, of Monmouth, £1,000 is left to the National Lifeboat Institution for a boat to be stationed at the mouth of the river Usk, and one-third of the residue of his estate is to be divided between the Newport Infirmary, the Bristol Eye Hospital, and the General and Eye Hospital, Swansea.

Mr. A. H. Lloyd, of Bletchingley, Surrey, left £232,690, and bequeathed £1,000 each to King Edward's Hospital Fund, Guy's Hospital, North-West London Hospital, Chelsea Hospital for Women, St. Peter's Hospital, East London Hospital for Children, London Hospital, Charing Cross Hospital, and the Samaritan Free Hospital; £500 each to the West London Hospital, the Royal Hospital for Incurables, the British Home for Incurables, the National Hospital for the Paralysed and Epileptic, Croydon Hospital, Central Throat and Ear Hospital, the North-Eastern Hospital for Children; and legacies to a number of other institutions.

